# A Novel Triazole Schiff Base Derivatives for Remediation of Chromium Contamination from Tannery Waste Water

**DOI:** 10.3390/molecules27165087

**Published:** 2022-08-10

**Authors:** Ahmad A. Alluhaybi, Ahmed Alharbi, Ahmed M. Hameed, Ayman A. Gouda, Fatma S. Hassen, Hassan S. El-Gendy, Bahig M. Atia, Amany R. Salem, Mohamed A. Gado, Antoaneta Ene, Hamdy A. Awad, Hesham M. H. Zakaly

**Affiliations:** 1 Department of Chemistry, College of Science and Arts, King Abdulaziz University, Rabigh 22254, Saudi Arabia; 2 Department of Chemistry, Faculty of Applied Sciences, Umm Al-Qura University, Makkah 21955, Saudi Arabia; 3 Chemistry Department, Faculty of Science, Zagazig University, Zagazig 44519, Egypt; 4 Nuclear Materials Authority, P.O. Box 530, El Maadi, Cairo 11936, Egypt; 5 INPOLDE Research Center, Department of Chemistry, Physics and Environment, Faculty of Sciences and Environment, Dunarea de Jos University of Galati, 47 Domneasca Street, 800008 Galati, Romania; 6 Geology Department, Faculty of Science, Al-Azhar University, Assiut Branch 71524, Egypt; 7 Institute of Physics and Technology, Ural Federal University, Yekaterinburg 620002, Russia; 8 Physics Department, Faculty of Science, Al-Azhar University, Assiut 71524, Egypt

**Keywords:** schiff base, adsorption, triazole derivatives, chromium, tannery waste water

## Abstract

Tannery industries are one of the extensive industrial activities which are the major source of chromium contamination in the environment. Chromium contamination has been an increasing threat to the environment and human health. Therefore, the removal of chromium ions is necessary to save human society. This study is oriented toward the preparation of a new triazole Schiff base derivatives for the remediation of chromium ions. 4,4′-((1*E*)-1,2-bis ((1*H*-1,2,4-triazol-3-yl) imino)ethane-1,2-diyl) diphenol was prepared by the interaction between 3-Amino-1*H*-1,2,4-triazole and 4,4′-Dihydroxybenzil. Then, the produced Schiff base underwent a phosphorylation reaction to produce the adsorbent (TIHP), which confirmed its structure via the different tools FTIR, TGA, ^1^HNMR, ^13^CNMR, GC-MS, and Phosphorus-31 nuclear magnetic resonance (^31^P-NMR). The newly synthesized adsorbent (TIHP) was used to remove chromium oxyanions (Cr(VI)) from an aqueous solution. The batch technique was used to test many controlling factors, including the pH of the working aqueous solution, the amount of adsorbent dose, the initial concentration of Cr(VI), the interaction time, and the temperature. The desorption behaviour of Cr(VI) changes when it is exposed to the suggested foreign ions. The maximum adsorption capacity for Cr(VI) adsorption on the new adsorbent was 307.07 mg/g at room temperature. Freundlich’s isotherm model fits the adsorption isotherms perfectly. The kinetic results were well-constrained by the pseudo-second-order equation. The thermodynamic studies establish that the adsorption type was exothermic and naturally spontaneous.

## 1. Introduction

One of the most commonly detected toxins in the environment is Cr(VI), which is extremely hazardous to living things and may cause cancer in people. There are two types of chromium: Cr(VI) and (III). As a nutrient, Cr(III) is vital for animals, due to its ability to conserve active glucose, protein, and lipid metabolism [1]. In contrast, because it can permeate across cell membranes as CrO_4_^2−^ or HCrO_4_^−^, Cr(VI) is extremely harmful. The consumption of water contaminated with Cr(VI) poses health problems due to its toxicity [2]. The permissible limit concentration for Cr(VI) ions in drinking water is less than 50 µg/L, as recommended by the World Health Organization (WHO) and the United States Environmental Protection Agency (USEPA) [3,4]. In industrial operations such as the tanning of leather, electroplating, preservation of wood, dye manufacture, and the production of paint and paper, as well as petroleum refining activities, chromium is produced in significant quantities. Concentrations of Cr in states of (VI) and (III) in the effluents from these businesses range from tens to hundreds of milligrams per litre, depending on the process.

Skin irritation, lung tumours, and kidney, liver, and stomach damage are all possible side effects of Cr(VI), which is 500 times more dangerous in its hexavalent form than its trivalent counterpart [5].

The molecular systems that regulate lipid, glucose, and protein metabolism depend on Cr(III) and Cr(VI) regularly. Due to the unique chemical characteristics of Cr (III) and Cr (VI), the separation of the two Cr species has become extremely significant [6,7]. Metals can be identified with high precision and accuracy using flame atomic absorption spectrometry (FAAS). However, it lacks sufficient sensitivity to detect traces of components. An effective method of pre-concentration is therefore typically necessary. FAAS is unable to distinguish between distinct chemical species of analyses and can only detect the overall quantity of analyte. Chromate ion-selective sorption has been effectively achieved using flow injection (FI) through line column pre-concentration procedures that utilize solid phase extraction (SPE) [8]. Spectrophotometers are also one of the most essential tools for determining chromium with great precision and accuracy.

Chemical precipitation, ion exchange redox, and adsorption have all been used in the past to remove Cr(VI). When it comes to removing Cr(VI) from industrial effluent, the most successful and straightforward procedure is adsorption [9,10]. The adsorption technique requires an inexpensive adsorbent, has a high adsorption capacity, and is readily available. Other management approaches, for example, adsorption membrane, reduction, bioremediation, and photocatalytic, have been advanced to counteract the disadvantages of the precipitation process [11,12,13]. Adsorption has become more popular due to its wide variety of applications, high efficiency, low cost, and remarkable reproducibility [14,15,16,17]. The contact between the active groups (for example, carboxyl, ammonium groups, and hydroxyl) in the adsorbents and the pore volumes of those materials during the physical adsorption phase of the process is critical to the elimination of Cr(VI) [18,19]. Carbon materials have become a good choice for adsorbents [20,21,22] because they have a lot of pores, a lot of active groups, strong properties, and good thermal stability.

Chemical interaction between any amine of primary type and a ketone or an aldehyde under mild conditions produces Schiff bases, commonly known as imines, which have an imine or azomethine (C=N) group. They can be used in a variety of industries, including the production of thermally resistant, semi-conductive, and anticorrosive materials. Due to their antimicrobial, antimalarial, and antibacterial characteristics, the synthesis of Schiff bases has been highlighted in biological simulations, the design of molecular magnets, and the production of pharmaceuticals [23,24,25]. Schiff bases are very important in coordination chemistry due to their ability to act as donors.

To date, Schiff bases have been one of the most popular and thoroughly studied molecular chemo-sensors for choosy sensing of metal ions due to their simplicity of synthesis as well as their good biological activity and strong photophysical characteristics [26,27,28]. Schiff bases with something like a C=N bridge can easily isomerize in the excitation state and have extremely weak fluorescence because of the C=N isomerization [29,30].

However, when this efficient group can coordinate and form a complex with metal ions, the C=N isomerization is prevented, resulting in considerably brighter fluorescence signals. Due to their ease of synthesis, electrical characteristics, and high solubility, Schiff base ligands have been widely explored in coordination chemistry. The stability of these imine ligands under a wide range of oxidative and reductive conditions shows the border between hard and soft Lewis bases, which provides a borderline. Biochemical, analytical, and antibacterial reagents are becoming increasingly significant [31,32].

In this paper, the 3-Amino-1*H*-1,2,4-triazole and 4,4’-Dihydroxybenzil were used as the starting material to synthesise a new Schiff base structure, TIHP. The new Schiff base structure was then subjected to a phosphorylation reaction to increase the adsorption capacity. Scanning electron microscopy (SEM), Fourier transform infrared spectrometer (FT-IR), Brunauer-Emmett-Teller specific surface area analyzer (BET), solid-state ^13^C nuclear magnetic resonance (^13^CNMR), and X-rays were used to figure out the physical and chemical properties of the new adsorbent materials, TIHP. The adsorption performance of the TIHP materials was tested by static adsorption experiments.

## 2. Materials and Methods

### 2.1. Chemicals Utilized and Used Reagent

The aluminium chloride (AlCl_3_) and sodium hydroxide were supplied by Scharlau Chemie Company S.A. (Barcelona, Spain), 3-Amino-1*H*-1,2,4-triazole was purchased from Thermo Fisher Scientific-Acros Organics Inc. ((Morris Plains, NJ, USA), 4,4’-Dihydroxybenzil and DMF were obtained from Fluka (Charlotte, NC, England). Ethanol, isopropanol and phosphoryl trichloride were obtained from Sigma-Aldrich (St. Louis, MO, USA). Prior to usage, the solvents were purified and decontaminated using fixed scientific techniques and then newly distilled to ensure extreme effectiveness. All procedures were carried out in flame-dried glassware. The progress of the reaction was detected using thin paper chromatography (PC). The mixture of (ethyl acetate + ethanol 50:50 *v*/*v*) as an eluent has been used as spots on the thin paper chromatography plates which have been identified with a simple UV lamp at 250 nm.

### 2.2. Equipment

FTIR and SEM were applied to examine the new adsorbent (a Hitachi S-4160 scanning electron microscope). The concentration of metal ions was determined via inductively coupled plasma (ICP-AES) (Varian liberty 150 XL).

The Perkin-Elmer Frontier was applied to take FT-IR spectra. Dimethyl sulfoxide (DMSO) or chloroform (CDCl_3_) were applied as solvent materials for routine spectra of NMR at ambient temp. on an Avance TM 400 spectrometer. It is important to note that all chemical modifications are stated in ppm δ relative to the trace resonance of protonated chloroform, CDCl_3_ dimethyl sulfoxide, DMSO, and external 85% aqueous H_3_PO_4,_ as (*δ* 7.25 ppm), (*δ* 77.0 ppm), or (*δ* 2.50 ppm), (*δ* 39.51 ppm) and (*δ* 0.0 ppm), respectively.

A mass spectrum was examined by using a GC Finnigan MAT SSQ-7000 type of mass spectrometer. The progress of the reactions and examination of the compound’s purity was completed. The latter was done via thin Layer Chromatography (TLC) on silica gel-precoated aluminium sheets (Type 60, F 254, Merck, Darmstadt, Germany) with an eluent of petroleum ether (60–80 °C)/ethyl acetate, and the spots were identified by exposure to UV light at a lamp at λ_254_ nm for several seconds. The chemical names of the synthesized chemicals are designated using the IUPAC nomenclature. Conventional drying and purification processes were utilized.

### 2.3. Batch Adsorption

The standard solution of 1000 mg/L of chromium was made via dissolving 2.82 g of K_2_Cr_2_O_7_ in 1 L of distilled water and then diluting it. The dilution principle was used to sequentially dilute this stock.

The aqueous solution was reserved in interaction with a suitable dosage of TIHP in an Erlenmeyer (100 mL) in a shaker-incubator instrument type (FTSH-301 MINI (SP)) at 150 rpm for the batch-mode adsorption tests. Before we start the experimental adsorption studies, we start studying the best-controlling factors affecting the adsorption of Cr(VI), each separately, a single experiment for adsorption of Cr(VI) using 0.1 g/L TIHP, in the pH range of 2–7 with an initial Cr(VI) concentration that is 250 mg/L, shaking for 30 min at 25 °C temperature.

When adjusting the pH, NaOH and HCl solutions were used. The second series of adsorption studies were done in the dosage variety of 0.01–0.25 g/L at the best pH to find the optimum adsorbent dose. There were no new operating settings in this series of tests. Temperatures of 25 °C, initial concentration of 250 mg/L, and time intervals of 5 min to 90 min were used in the kinetic tests. The starting concentration of Cr(VI) was ranging from 250 to 1500 mg/L during the equilibrium trials. The adsorption kinetics were used to determine the equilibrium time. Adsorbents were tested for their ability to adsorb Cr(VI) and another interfering ion in different binary adsorption studies. The final Cr(VI) concentrations were determined by atomic absorption spectroscopy (Varian AA240) and UV-VIS spectroscopy type (Rigol-Ultra-3660 spectrophotometer at the extreme wavelength (λ = 590 nm)) in the multicomponent adsorption studies. Every experiment was attended twice and the average values were provided. Here is how the adsorption capacity and efficiency of Cr(VI) ions were considered using equations (1) and (2), which were used to mathematically calculate the adsorption capability (q_t_ (mg/g)), as follows:

(1) $$q_{t}=\frac{\left(C_{o}-C_{t}\right)V}{m}$$

(2) $$S\%=\frac{C_{o}-C_{e}}{Co}\times 100$$

For a given pollutant concentration, C_o_ (mg/L) is the starting pollutant concentration, C_t_ (mg/L) is the pollutant concentration at a specified time, C_e_ (mg/L) is the equilibrium pollution concentration, V is the volume of solution (L), and m denotes the mass of adsorbent (g).

### 2.4. Determination of Cr(VI) Concentration

The content of Cr(VI) was determined using the 1,5-diphenylcarbazide method [33], which involved determining the absorbance of each sample at (540 nm) with a Hitachi U-2900 UV-Vis spectrophotometer equipped (Tokyo, Japan).

To determine the presence of chromium Cr(VI), the certified analytical methods of the Society of Leather Technologists and Chemists [34,35] were followed. The material (25 mL) was placed in an Erlenmeyer flask (250 mL) and treated with HNO_3_ (15 mL), followed by an HClO_4_/H_2_SO_4_ mixture (15 mL). After that, the mixture was slightly heated until it turned a bright orange-red color, after which it was left to boil for another minute. This was followed by eliminating the flask from the heat source and fast cooling it in a cold water bath while it was whirling around. Following that, 75 mL of distilled water was wisely added along with a few glass beads (anti-bumping granules) and the mixture was brought to a boil for 10 min to eliminate any remaining free chlorine. Slowly, 10 mL of 30 percent H_2_SO_4_ was added to the liquid, which was then cooled to 20 °C. The working solution was titrated with newly made ferrous ammonium sulphate in which N-phenyl anthranilic acid acted as an indicator. It was signified by a color shifted from a violet color to a green one, and then the Cr(VI) concentration was mathematically calculated.

### 2.5. Preparation of TIHP

At first, 3-Amino-1*H*-1,2,4-triazole (0.1 mol/L) was measured in a 250 mL three-neck flask. Then, 60 mL of ethanol was added and stirred until 3-Amino-1*H*-1,2,4-triazole was completely dissolved. A 30 mL ethanolic solution of 4,4’-Dihydroxybenzil (1 mmol, 1.02 g) was added to the ethanolic solution of 3-Amino-1*H*-1,2,4-triazole. The mixture was stirred to reflux at 150 ℃ for 5 h. The precipitate was filtered off and recrystallized from the mixed solution of ethanol and isopropanol to obtain a pale yellowish precipitate formed and 4,4’-((1*E*)-1,2-bis((1*H*-1,2,4-triazol-3-yl)imino)ethane-1,2-diyl)diphenol was obtained.

**Scheme 1:**
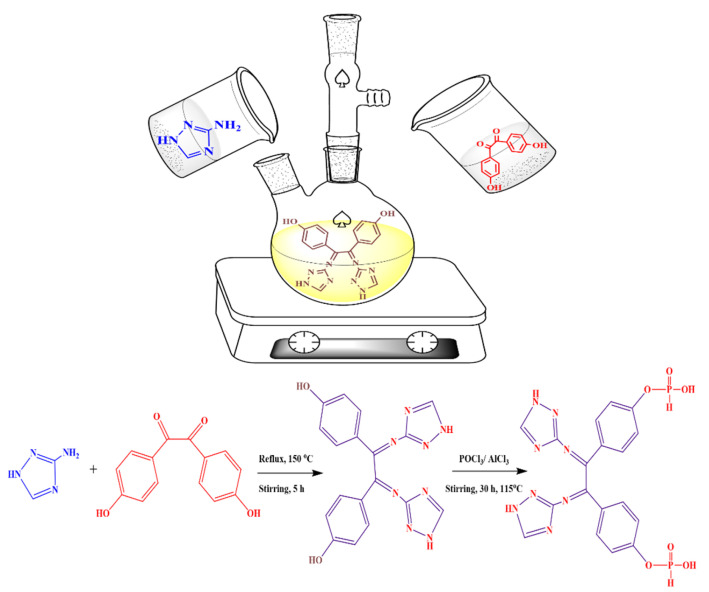
Preparation of TIHP.

The produced Schiff base was then subjected to phosphorylation reaction via interaction with 0.15 g AlCl_3_ (anhydrous) with 20 mL phosphorus oxychloride for 30 h at the temperature of 115 °C. A tail gas absorber to absorb HCl in the reaction process was installed. Then, in an ice bath, 200 mL of distilled water was gently added to the resulting mixture slowly drop by drop. The working mixture was filtered and drained with distilled water several times and dried in a vacuum at 80 °C for 30 h. Finally, a white precipitate of 4-((1*E*)-1,2-bis((1*H*-1,2,4-triazol-3-yl)imino)-2-(4-((hydroxyhydrophosphoryl) oxy) phenyl) ethyl) phenyl hydrogen phosphonate TIHP was obtained. The synthesis process is shown in Scheme 1.

FTIR (Nicolet 6700 spectrometer), mass spectra, ^13^C analysis, and ^31^P NMR studies were used to characterize the produced product. A Bruker instrument, AVANCE III HD 400, was used for the NMR analysis.

## 3. Results and Discussions

### 3.1. Characterizations of TIHP

#### 3.1.1. FTIR Analysis of TIHP

**Figure 1:**
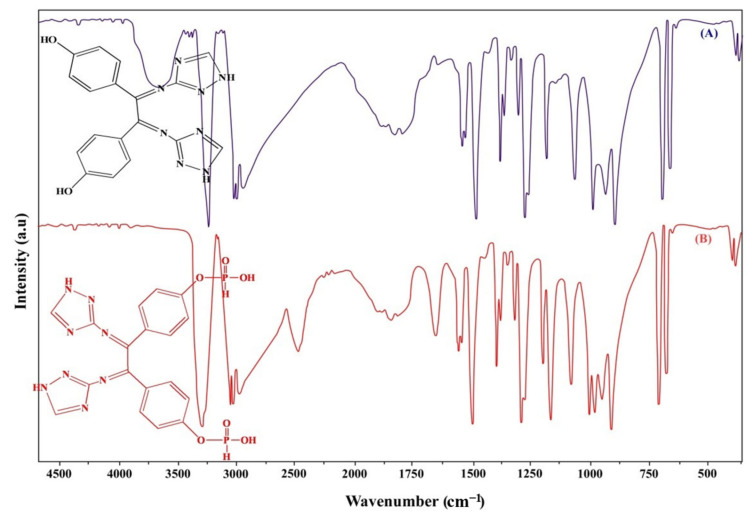
FTIR spectrum of (**A**) TIH, (**B**) TIHP.

A Schiff base imine group assignement of TIH was located at 1636 cm^−1^ [36]. There are fresh new peaks at 1517 cm^−1^, 1498 cm^−1^, 756 cm^−1^, and 1284 cm^−1^, which correspond to the C-C bonds on the benzene ring of Dihydroxy Benzil in TIH. The benzene ring’s stretching and bending vibration peaks, as well as the C-O ring’s stretching vibration peak on phenol [37], are noted. The peaks at 1638 cm^−1^ and 1595 cm^−1^ were found to correspond to the amide group and to -NH_2_ symmetrical stretching vibration, respectively [38]. To confirm the phosphorylation reaction’s chemical structure, the P-H stretching vibration (2350 cm^−1^), P=O stretching vibration (1100 cm^−1^), P-OH stretching vibration (991 cm^−1^), and the peak detected at 874 cm^−1^ are all FTIR bands that correspond to the P-O-C bending vibration [39], as can be seen in Figure 1.

#### 3.1.2. The Brunauer-Emmett-Teller Analysis of TIHP

**Figure 2:**
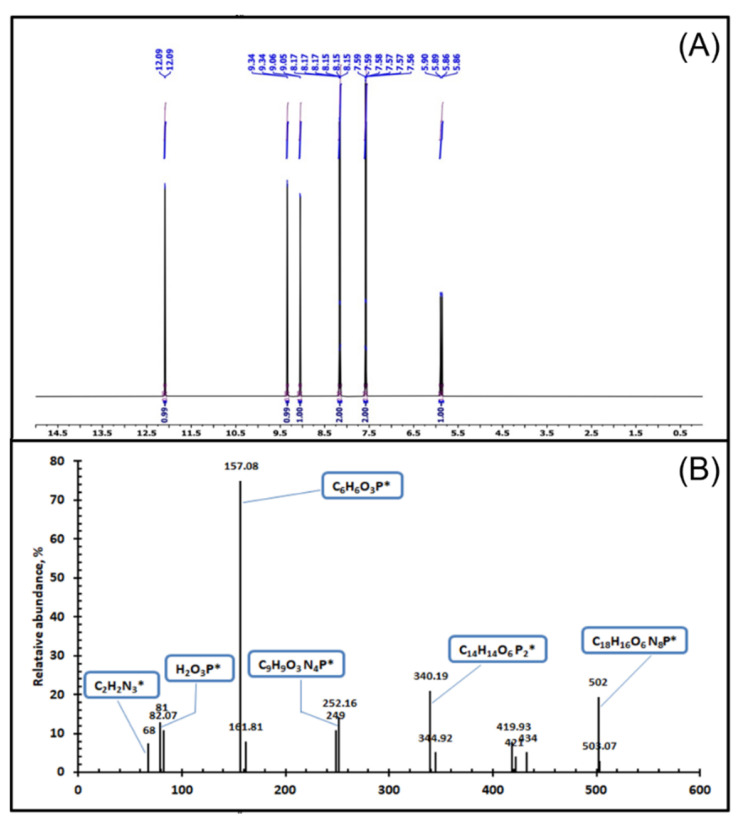
(**A**) ^1^H-NMR analysis of TIHP. (**B**) GC-Mass analysis of TIHP.

Adsorption/desorption of N_2_ was performed at 77 K which was used to determine the TIH and TIHP surface areas (S_BET_) and porosity. Reversible isotherms, as seen in Figure 2, are characterized by a significant rise in uptake when a relative pressure is low; this indicates that the TIHP has both micro- and mesopores, as seen in Figure S1. The IUPAC classifies the nitrogen sorption isotherms in the TIH and TIHP as type IV. Conferring to Figure 2A,B, TIHP contains an H3 hysteresis loop isotherm and no clear saturation adsorption platforms, which suggests that the pores are quite irregular. Both TIH and TIHP have Brunauer-Emmett-Teller surface areas of 37.2 and 68.4 m^2^∙g^−1^, respectively, with total volumes of 0.189 and 0.143 cm^2^∙g^−1^, as seen in Table 1.

#### 3.1.3. H-NMR Analysis

^1^H-NMR (400.15 MHz, CDCl_3_, 25 °C, TMS) δ, ppm: 12.09 (d, *J* = 1.4 Hz, 2H, -NH group), 9.34 (d, *J* = 1.3 Hz, 2H, -CH of triazole ring protons), 9.05 (d, *J* = 3.3 Hz, 2H, -OH group), 7.58–8.17 (d, 8H, benzene ring), 5.88 (d, *J* = 14.3 Hz, 2H, -PH group). ^1^H-NMR analysis with the energy of 400.15 MH_Z_ and CDCl_3_ as a diluent is an operational and cooperative tool which gives significant data about protons in the synthesized triazole derv. ligand which contributes to the structure prediction. The main δ (ppm) assignments appear at 12.09 ppm which is related to the more de-shielded protons of the NH group. It was observed that the assignment of -OH protons (δ = 9.05 ppm) is also de-shielded but less than -NH protons (δ = 12.09 ppm). The two assignments of benzene ring protons and the -PH group were noticed at a chemical shift of 7.58–8.17 and 5.88 ppm, respectively. The characterization of triazole derivatives and chelating ligand using ^1^H-NMR are shown in Figure 2A.

#### 3.1.4. GC/MS Analysis

GC/MS (EI, 30 eV), *m*/*z* (% rel): [*m*/*z*]^+^ of 502, 68, 157, 81 , 340, 252 and 82. Anal. Calc. for C_18_H_16_N_8_O_6_P_2_ (502.32 g/mol): C, 43; H, 3.21; N, 22.31; P, 12.33; O, 19.12. Found: C, 43.01; H, 3.2; N, 22.33; P, 12.35; O, 19.22. Gas chromatography with a mass spectrometer unit (GC/MS) was demonstrated to be a powerful and commanding tool for predicting molecular formula, purity, and the more stable fragment [*m*/*z*]^+^. Some significant fragmentation patterns are associated with the synthesis of triazole derv. The chelating ligand was shown such as [C_2_H_2_N_3_] with a molecular weight of 68 (triazole ring), [C_6_H_6_O_3_P] with a molecular weight of 157 (benzo phosphonate moiety), [H_2_O_3_P] with a molecular weight of 81, which signifies the phosphonate radical and [C_18_H_16_N_8_O_6_P_2_] with a molecular weight of 502 which characterizes the molecular ion peak of triazole derv. chelating ligand. The whole analysis achieved assures a satisfactory synthesis of the ligand. A description of triazole derv. using GC/MS is presented in Figure 2B.

#### 3.1.5. ^13^C-NMR Analysis

^13^C-NMR (100.05 MHz, CDCl_3_, 25 °C, TMS) δ, ppm: 163.2 (s, *J* = 15.1 Hz, 2C, aliphatic carbon), 144.4–158.7 (s, *J* = 3.5 Hz, 4C, triazole ring), 120.7–156.5 (s, *J* = 7 Hz, 12 C of benzene ring). ^13^C-NMR analysis with an energy of 100.05 MH_Z_ and CDCl_3_ as a diluent is an operational technique which gives important data about the number of carbon atoms in the prepared ligand. The main δ (ppm) appeared around 120.7–156.5 ppm which is related to a carbon atom of a benzene ring. The aliphatic carbon atoms which are located in the middle of the ligand have an assignment of 163.2 ppm with a coupling constant of J = 15.1 Hz. A distinct assignment was established to be more de-shielded carbon, which appears between 144.4 and 158.7 ppm which is related to the triazole ring carbon. Specification of triazole derv. chelating ligand using ^13^C-NMR is shown in Figure S2.

#### 3.1.6. P-NMR Analysis

^31^P-NMR analysis with the energy of 151 MH_Z_ and CDCl_3_ as a diluent is an active and supportive tool to identify the phosphorus atom in the prepared triazole derv. chelating ligand. The main δ (ppm) assignment appears as a doublet at 5.2 and 5.3 ppm with a coupling constant J = 15 Hz. The presence of two assignments and a higher coupling constant value may indicate that there is an asymmetry in the ligand as a result of its rotation. Chart of the triazole derv. chelating ligand using ^31^P-NMR is shown in Figure S2.

#### 3.1.7. Thermal Analysis

**Figure 3:**
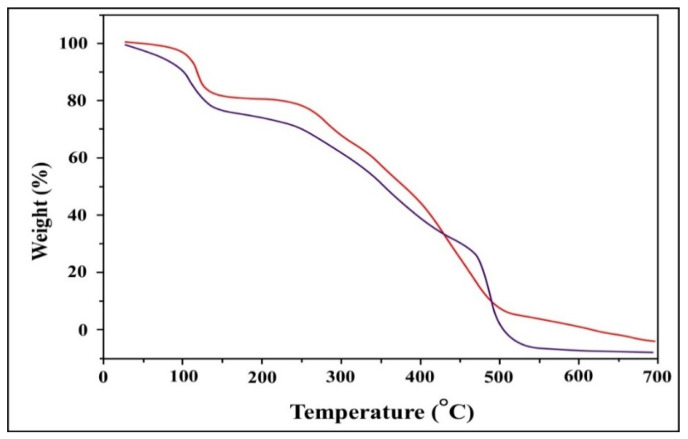
TGA curve of TIH (blue) and TIHP (red).

The TGA curve for TIHP (RED) (Figure 3) shows a mass loss at 25–121 °C because of the loss of surface water molecules, the 2nd mass loss was observed at 122–237 °C because of the loss of coordinated water molecules or crystal lattice water molecules, and loss of NH_3_ [40]. The 3rd loss was observed at a temperature range of 238–500 °C which can be attributed to the evolution of the Schiff base ligand as CO and CO_2_ and creating carbonaceous waste. The last mass loss was observed at a temperature range of 500–700 °C due to the decomposition of the phosphonic acid group [41].

The TGA curve for TIH (blue). The loss of adsorbent moisture can be attributed to the first weight loss noticed below 100 °C, the 2nd one is assigned for the loss of coordinated water molecules or crystal lattice water molecules. While the latter ranges from 220 to 500 °C and can result in NH_3_ loss, thermal degradation of the Schiff base ligand, CO and CO_2_ evolution, [42] and the formation of carbonaceous waste [43].

### 3.2. Factors Controlling the Adsorption Process

Numerous parameters have been investigated, including the pH of the Cr(VI) solution, the TIHP dose, the time of interaction, the initial concentration, and the temperature.

#### 3.2.1. The Effect of pH

The sorbent surface’s acid-base properties are significantly influenced by pH, and this in effect has a major impact on the adsorption effectiveness. The higher quantity of H^+^ ions formed at low pH values (pH = 2) led to superior chromium (VI) adsorption capability. Chromium anionic forms, such as Cr_2_O_7_^2−^, HCrO_4_^−^, or CrO_4_^2−^ can be produced in solutions with pH values ranging between 2 and 12 [44]. When pH rises to 6, on the other hand, the extra OH ions in the solution raise the hindrance to dichromate ion diffusion [45].

The metal ion sorption progression is influenced by the solution’s pH. As a part of this study, the influence of pH on the elimination of Cr(VI) was examined, and up-taking adsorption of Cr(VI) by TIHP was explored using 0.1 M NaOH /HCl to adjust the solution pH from 1 to 7. The efficient groups on the TIHP surface are involved in the adsorption of Cr(VI). TIHP function groups are reactive to hexavalent chromium. On the basis of pH, it is clear that the various oxyanions of chromium have established an equilibrium in water [46].

$$\;\;\;H_{2}{\mathrm{CrO}}_{4}\to H^{+}+{\mathrm{HCrO}}_{4}^{-}\;{\mathrm{HCrO}}_{4}^{-}\to H^{+}+{\mathrm{CrO}}_{4}^{2-}\;\;\;\;\;\;\;2{\mathrm{HCrO}}_{4}^{-}\to {\mathrm{Cr}}_{2}O_{7}^{2-}+H_{2}O$$

Chromium is classified as a hard acid, and nitrogen is classified as a hard base, that is according to Pearson’s idea of hard and soft acids and basic principles. Cr(VI) occurs as HCrO_4_^−^ (hydrogen tetraoxochromate anion) in the pH between 2 and 4, while CrO_4_^2−^- (tetraoxochromate oxyanion) dominates at higher pH dichromate ion Cr_2_O_7_^2−^ predominates in strongly acidic solutions (pH less than 2) [47].

**Figure 4:**
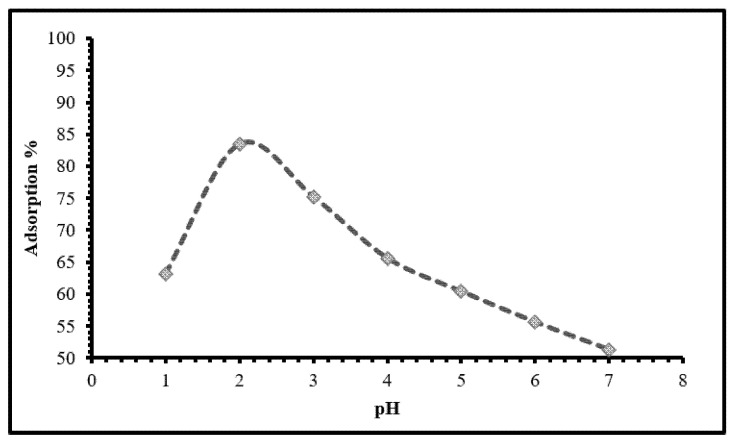
Influence of pH for the up taking of Cr(VI) using TIHP, (time 30 min., 20 mL sample, 0.1 g TIHP, T = 25 ± 1 °C and [Cr(VI)] = 250 mg/L).

The pH range of 2–4 was shown to be optimal for hexavalent chromium adsorption on TIHP adsorbent, as seen in Figure 4. It is quite probable to visualize a chelation mechanism through the long pairs of electrons of a phosphate group and lone pair of oxygen with Cr(VI). Furthermore, chelation between Cr(VI) ions and lone pair of electrons of the nitrogen atoms of triazole can be observed [48,49,50]. A decrease in the adsorption percentage occurs as the repulsion between the surface negatively charged TIHP and the hydrochromate anion weakens with rising pH. The TIHP surface charge converts negative with the gentle de-protonation of the surface hydroxy groups, producing electrostatic repulsion between the hexavalent chromium oxy anion in alkaline pH, as seen in Figure 4 [45].

**Figure 5:**
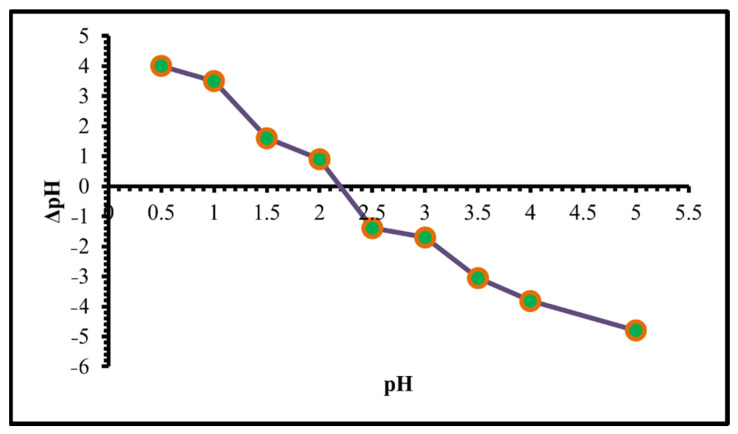
pH point of zero charges of TIHP.

The pH of zero point of charge (pH pzc) or the isoelectric point is the point at which the adsorbent surface charge becomes zero or the pH value at which the surface anionic and cationic charges of the TIHP are equal. When the pH < pH_zpc_, the TIHP surface turns cationic due to protonation. On the other hand, when the solution pH > pH_zpc_, the surface is negative which cationic ions prefer for adsorption [51,52]. The pH_pzc_ value for TIHP is 2.2. which indicates that the TIHP adsorbent surface acts as cationic due to protonation, as seen in Figure 5.

#### 3.2.2. Influence of Adsorbent Dosage

Cr(VI) sorption at pH 2 was tested with various adsorbent dosages, and it was discovered that increasing the adsorbent dose improved the total surface area presented for a representative adsorption process, making it a useful parameter for this type of procedure. Increasing the number of adsorbents improves both the number of active sites and the percentage of adsorption that can be achieved. When the driving force goes down, some of the active sites on an adsorbent become full, and the adsorption capacity goes down.

**Figure 6:**
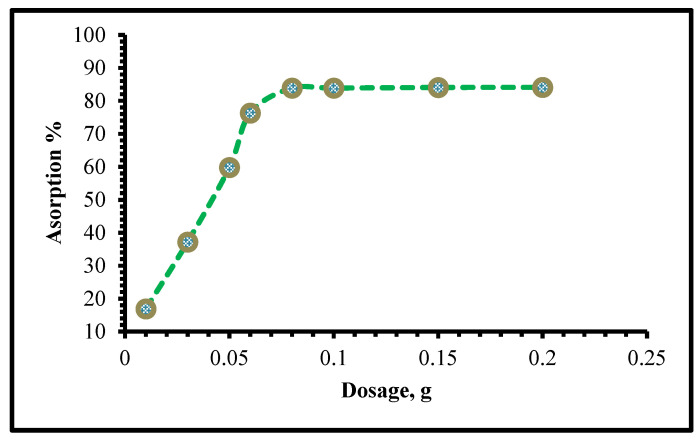
Influence of adsorbent dosage upon the adsorption capability of Cr(VI) on TIHP (time 30 min, 20 mL sample, pH 2, [Cr(VI)] = 250 mg/L, and T = 25 ± 1 °C).

According to Figure 6, increasing the adsorbent dosage from 0.01 to 0.06 g improved the Cr(VI) adsorption efficiency from 16.9 to 68.3%, achieving the maximum adsorption rate at an adsorbent dosage of 0.08 g, which was chosen as the optimal up-taking dosage for the Cr(VI) adsorption process.

#### 3.2.3. Effect of Exchange or Contact Time

**Figure 7:**
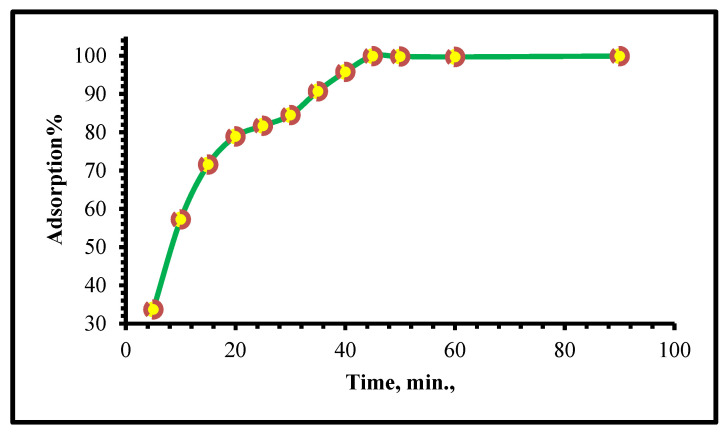
Influence of contact time upon the Cr(VI) adsorption using TIHP. [Cr(VI)] = 250 mg/L, TIHP dose = 0.08 g, pH = 2, T = 25 ± 1 °C.

In Figure 7, the Cr(VI) adsorption capacity is shown as the meaning of the interaction time for TIHP adsorbent at a pH value of 2 and an initial Cr(VI) concentration of 250 mg/L. The results indicate that the equilibrium is achieved within 40 min. Furthermore, it is marked that the uptake of Cr(VI) is fast at the beginning; more than 75% of the initial Cr(VI) quantity is adsorbed after 20 min, while at equilibrium, the rest of the Cr(VI) percentage is adsorbed so that from Figure 7 the equilibrium time for this study is 45 min.

#### 3.2.4. Adsorption Kinetics

Adsorption kinetics is critical since it sheds light on the reaction routes and mechanisms. Pseudo-first and second-order models were applied to study the uptake kinetics of Cr (VI) on THIP [53,54]. Approximately, 0.08 g of TIHP adsorbent was added to 250 mg L^−1^ Cr(VI) concentration (20 mL) for analysis. In this case, we lowered the original pH to 2. At a constant agitation speed of 150 revolutions per minute (RPM), the concentrations of Cr(VI) solution phase were measured at intervals of 5–90 min. Linear pseudo-first-order and second-order models were used to study the kinetics of Cr(VI) adsorption. The pseudo-first-order equation can be stated as follows [55]:

(3) $$\log(q_{e}-q_{t})=\log q_{e}-\frac{K_{1}t}{2.303}$$

The pseudo-second order equation is normally stated as:

(4) $$\frac{t}{q_{t}}=\frac{t}{q_{e}}+\frac{1}{k_{2}q_{e}^{2}}$$

The intraparticle diffusion model is expressed as:

(5) $$q_{t}=k_{\mathrm{dif}}.t^{0.5}+C$$

where q_e_ and q_t_ are the equilibrium and time-dependent amounts of Cr(VI) adsorbed (mg/g), and k1 is the first-order absorption rate constant (min^−1^).

**Figure 8:**
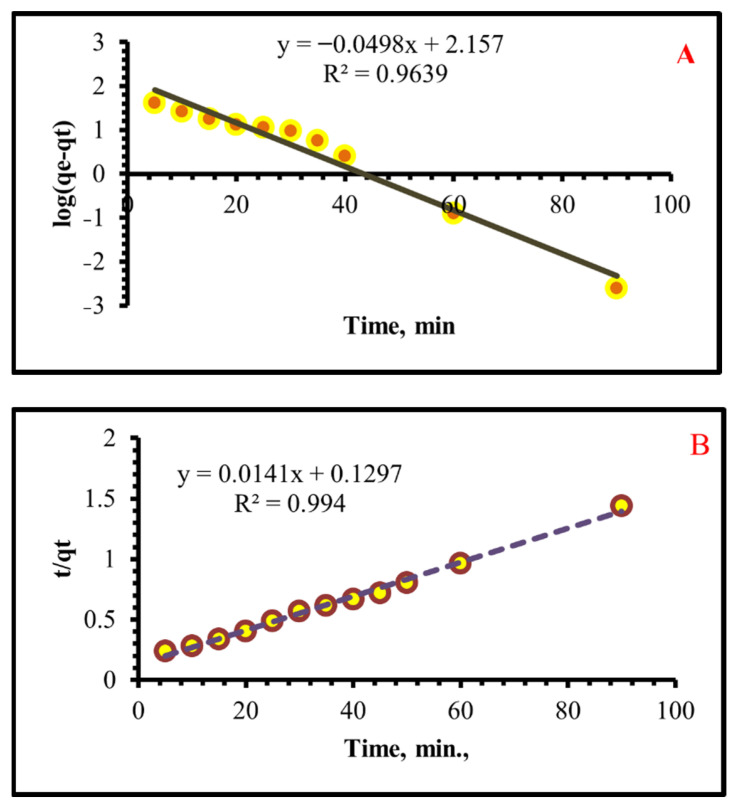
(**A**) Pseudo first-orders and (**B**) Pseudo second-order kinetics models of the adsorption of Cr(VI) upon TIHP at 25 °C; pH 2, T = 25 ± 1 °C and 250 mg/L initial concentration.

Figure 8 shows a plot of “log (q_e_ − q_t_)” vs “t” at various initial Cr(VI) concentrations. The pseudo-first-order constant is 0.11469 min^−1^. The calculated q_e_ values attained from the pseudo-first-order model do not agree with the experimental q_e_. The calculated equilibrium uptake capacity increases as the initial Cr(VI) concentration increases. The correlation coefficient (R^2^) at numerous concentrations is small for the kinetic model of the pseudo-first order.

As soon as sorption data are collected, it begins to vary from a straight line after around 25 min. This graph shows that the early adsorption obeys first-order kinetics, but later on it deviates from the first-order kinetics. The R^2^ values indicate that the adsorption data fit poorly to pseudo-first-order kinetics even though the q_e_(cal) and q_e_(exp) values are close. There is no diffusion control on the uptake of Cr (VI) onto the surface of the TIHP.

From the linear plots of log (q_e_ − q_t_) vs. time for pseudo-first order (Figure 8a) or (t/q_e_) vs. time for second-order (Figure 8b), the kinetic factors that control the two models can be found. The applicability of each model is tested by the appropriateness of the straight line (R^2^). A straight line is used as a test of each model’s validity (R^2^). Suppository first and second-order rate constants were provided in Table 2, along with equilibrium sorption capacities. The pseudo-second-order model is better adapted for the adsorption process than the other model of pseudo-first order, as shown in Table 2 for the TIHP adsorbent and the consistency of the predicted value and experimental value of q_e_. Adsorption follows a pseudo-second-order process and depends on the amount of Cr(VI) ions and the texture of the resin.

The intraparticle diffusion model discovered a relationship between Cr(VI) concentration and the square root of time (Fickian diffusion law [56]; qt = Ki t^0.5^ + C,), which is not linear, as seen in Figure S3. This means that the adsorbent’s overall adsorption rate is regulated by this factor, namely, interparticle diffusion and the diffusion of the boundary layer. qt against t^0.5^ crosses the origin when intraparticle diffusion is the only rate-limiting step. During film diffusion, the intercept is C, which indicates the thickness of the boundary layer’s film layer. From Figure S3, it is clear that the adsorption process was two steps. Intraparticle diffusion is shown by the next straight line after boundary layer diffusion. [56].

#### 3.2.5. Influence of Adsorption Temperature

**Figure 9:**
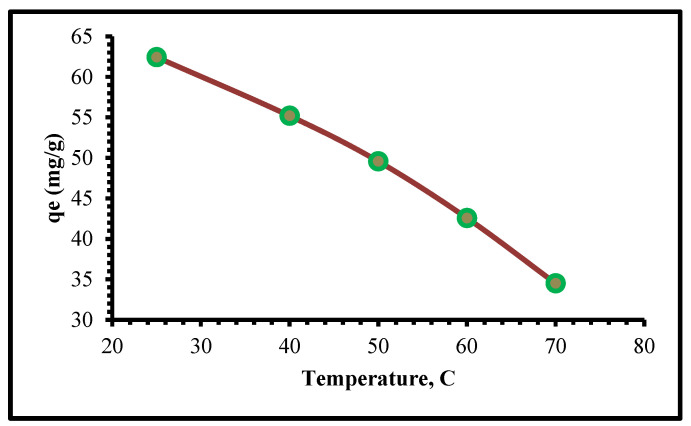
Effect of temperature on the adsorption of Cr(VI) upon TIHP.

Adsorption temperature experiments were analyzed in this routine: 0.08 g of TIHP was tested with a 250 mg/L Cr(VI) solution at a pH of 2 and temperatures ranging from 298 to 343 K. Figure 9 displays the estimated data. Experiential data show that as the temperature rises, the response rate decreases significantly. Since adsorption is an exothermic process, raising the temperature would not speed up the process.

#### 3.2.6. Effect of the Initial Concentration

**Figure 10:**
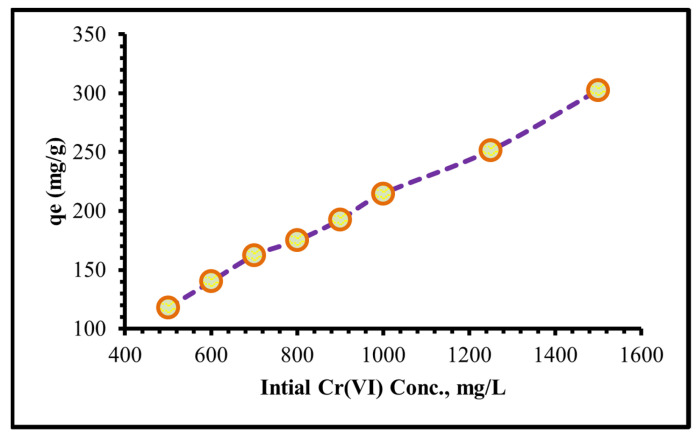
Influence of initial Cr(VI) concentration upon TIHP uptake.

At a pH of 2, initial concentrations of Cr(VI) solutions ranging from 250 to 1500 mg/L were extended, and the appropriate amount of adsorbent (0.08 g) was used in adsorption tests at 25 °C. As illustrated in Figure 10, the % of Cr(VI) that can be eliminated depends upon the concentration of Cr(VI). It was shown that as the starting concentration was raised, so was the adsorption effectiveness. The ratio of initial moles of Cr(VI) to accessible uptake surface area on TIHP increases with lower initial Cr(VI) concentration. The mass transfer driving forces increase as the initial concentration of Cr(VI) increases, resulting in significant Cr(VI) adsorption. As a result, an initial concentration of 1250 mg/L of TIHP adsorbent was found to be best in this experiment.

#### 3.2.7. Adsorption Isotherm Studies

The experimental Cr(VI) uptake data were examined using a variety of empirical isotherm models, including the Langmuir [57] and Freundlich [58] isotherms, which highlight several key features. In numerous adsorption experiments, linear isotherms have been extensively used. Due to some of the experimental data being nonlinear, the nonlinear model is a better fit for this particular study. This was accomplished by utilizing the nonlinear model to examine the Langmuir and Freundlich isotherm data, as well as the Origin 2020b software to get the required plots and isotherm parameter values. Table 2 lists the various isotherm parameters. The nonlinear Langmuir model yielded a q_max_ value of 320.23 mg∙g^−1^. (Table 3), Each of these isotherms can be fully described by looking at their plots and expressions.

(6) $$q_{e}=\frac{q_{m}K_{l}C_{e}}{1+K_{l}C_{e}}$$

(7) $$q_{e}=K_{f}C_{e}^{\frac{1}{n}}$$

**Figure 11:**
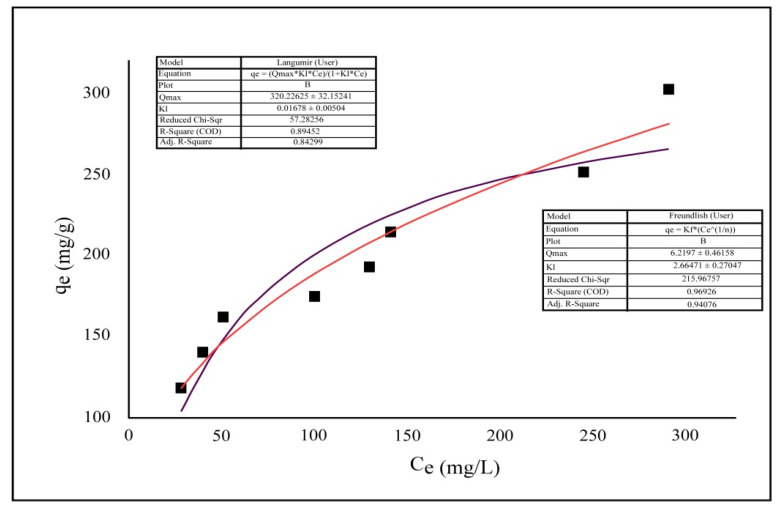
Non-linear adsorption isotherm models of Cr(VI) up taking upon TIHP fitted to experimental adsorption.

The obtained data illustrate the correlation coefficient (R^2^) of the Freundlich isotherm model is higher than that of the Langmuir model which means that the adsorption data of Cr(VI) ions onto TIHP adsorbent was best predicted by the Freundlich isotherm model, Figure 11.

#### 3.2.8. Adsorption Thermodynamics

Adsorption process feasibility and nature can only be determined with the help of a thermodynamics study. There are many thermodynamic parameters included, standard free energy symbols by (∆G°), standard enthalpy symbols by (∆H°), and standard entropy symbols by (∆S°). The changes were measured at temp. between 298 and 343 K.

**Figure 12:**
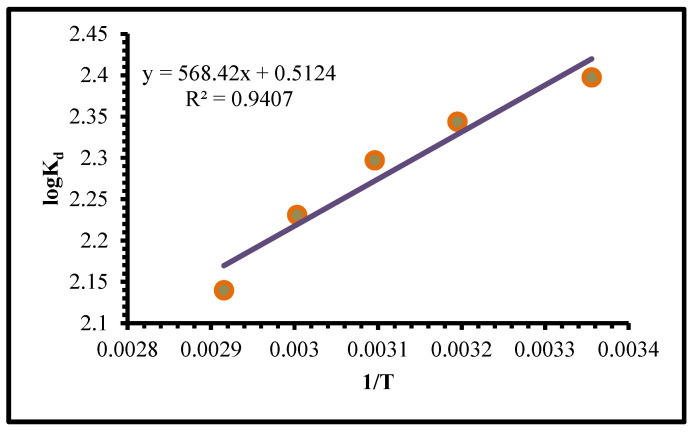
Plot of log K_d_ vs. 1/T of Mo(VI) adsorption on TIHP adsorbant.

Thermodynamic studies, which gives equilibrium constant, from the slope and intercept of the van’t Hoff plot of lnK against 1/T, ∆H° and ∆S° could be obtained (Figure 12), for the reaction of exothermic the slope is positive and also, the equilibrium constant reductions with rises in temp [59].

These thermodynamic factors were mathematically calculated via the following equation [60]:

(8) ΔG=ΔH − TΔS

(9) $$\mathrm{LogKd}=\frac{\Delta S}{2.303R}-\frac{\Delta H}{2.303\mathrm{RT}}$$

R = (8.314 J∙mol^−1^∙K^−1^) which is represented by the universal gas constant.

T is represented by the temp. in Kelvin (K). Figure 12 was used for the mathematical calculation of the ∆H and ∆S values from the slope and intersection of the Log K_d_ against the 1/T plot, which give a slope of 568.42 and an intersection of 0.5124 with a concerned correlation coefficient R^2^ = 0.9407. In Table 4, (−∆H) prove that Cr(VI) ion adsorption is exothermic, which means that heat is produced during the adsorption process.

The (+∆S) evidence of increased adsorption process variability was found while extracting. It was discovered that (−∆G) may be extracted spontaneously at low temperatures. The apparent activation energy (E_a_) for the extraction of Cr(VI) can be mathematically calculated using the Arrhenius equation by computing the slope of the straight line shown in Figure 12. It is possible to calculate the Arrhenius equation using the following equation [61,62]:

(10) $$LogK_{d}=\frac{-2.303E_{a}}{RT}+LogA$$

When calculating the molar gas constant (8.314 J/mol∙K^−1^), the partition coefficient K_d_, the extraction activation energy Ea (KJ/mol), the temperature in Kelvin K, and A standing for the pre-exponential component that is not dependent on temperature. Cr(VI) ions needed activation energy of −2.052 kJ/mol to be adsorbable. In other words, at room temp., the adsorption of Cr(VI) ions on TIHP is an exothermic reaction that does not require activation energy to occur.

#### 3.2.9. Effect of Foreign Ions

**Figure 13:**
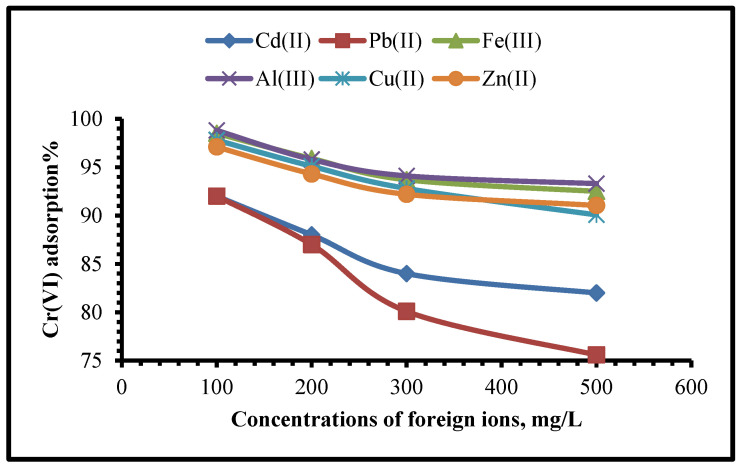
Influence of foreign cations upon the adsorption efficiency of Cr(VI) using TIHP adsorbant.

**Figure 14:**
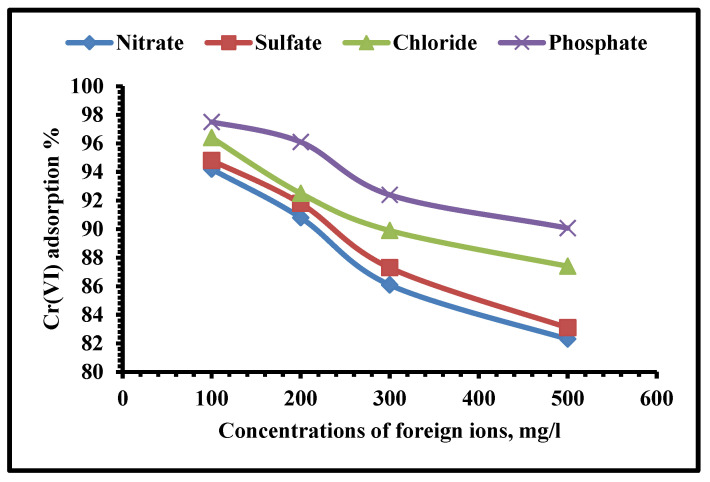
Influence of foreign anions upon the adsorption efficiency of Cr(VI) using TIHP adsorbent.

Cation and anion effects on the adsorption of Cr(VI) should be studied before practical applications of this approach can be developed. In order to conduct further tests, it was necessary to determine whether the presence of certain main ionic components could interfere with Cr(VI) adsorption. Through this approach, the concentration of the foreign (interfering) anion was ranging from 50 to 250 mg∙L^–1^. The interference of ions such as Pb^2+^, Cd^2+^, Zn^2+^, Cu^2+^, Co^2+^, Ni^2+^, Fe^2+^, and Mn^2+^ were studied individually in 100 mL sample volumes holding 250 mg·L^−1^ hexavalent chromium **(**Figure 13 and Figure 14**).**

##### Effect of Co-Existing Cations

It was found that the TIHP’s uptake capability for Cr(VI) was influenced by coexisting cations. Adsorption capability in the presence of 100–500 mg/L of interfering ions Cd^2+^, Pb^2+^, Cu^2+^, Fe^3+^, Zn^2+^, and Al^3+^, which demonstrated that the presence of Al^3+^ and Fe^3+^ only slightly reduced the adsorption capability of Cr(VI). These two cations (Al^3+^ and Fe^3+^) had an inconspicuous impact on Cr(VI) uptake. The uptake of Cr(VI) onto the TIHP was clearly influenced by the presence of the other four coexisting cations, with Pb^2+^ suffering the most as shown in Figure 13.

Although surface area and porosity were important factors in Cr(VI) absorption, surface-charged characteristics were the most important factor in Cr(VI) uptake. Because the modified adsorbent had a substantial number of positive charges on its surface, it was able to pull the chromium anions and reject the co-existing cations, the chromium anions could potentially interact with the cations to reduce removal effectiveness by a little amount. Furthermore, Sag et al. (2001) [62] found a link between the ionic radius of metal ions and the choosiness of the adsorbent for the concerning ion of Cr(VI).

The six metal cations that were under consideration are listed in detail in Table 5. Depending upon the work of Tobin et al. (1984) [63], the ionic radius and metal ion adsorption were found to be linearly connected.

HCrO_4_^−^ and Cr_2_O_7_^2−^ were the most common forms of chromium at pHs of 2.0–3.0 and 3.0–6.0, respectively^.^ It was noticed that Cr(VI) uptake was unfavourable at pH > 6.0 because CrO_4_^2−^ would compete with the OH- to positively charge the adsorption sites. It was shown that for pHs between 2.0 to 3.0 and 3.0 to 5.0, M^2+^ and M(OH)^+^ and M(OH)_2_ were the dominant metal cations and that M(OH)_2_ adsorption was larger than M(OH)^+^ on the non-polar surface. Metal ion adsorption is linearly linked to the ionic radius. The adsorbent’s ion-exchange efficiency was enhanced by a decrease in the hydration radius as the ionic radius increased. During the ion-exchange procedure, the Pb^2+^ cation with the biggest ionic radius may be easily attracted into the TIHP molecular structure. Consequently, the appearance of cations would compete with chromium for the adsorption sites with which they could interact, but also reduce the adsorption efficiency.

##### Effect of Co-Existing Anions

As shown in Figure 14, increasing the concentrations of competing anions (Cl^−^, and SO_4_^2−^) from 100 to 250 mg/L may have reduced Cr(VI) adsorption. Two factors are used to explain the decrease in the adsorption capacity of Cr(VI). The rate of diffusion to the THIP surface was limited by the migration of Cr(VI) in the solution. The 2nd factor is that the negatively charged Cr(VI) ions were adsorbed on the binding adsorption sites of positively charged THIP so that the coexisting anions inevitably competed with Cr(VI) [64].

According to surface chemistry theory, meanwhile, an electric double layer is formed with the adsorbate by a solid absorbent via electrostatic interaction [65], increasing the strength of coexisting ions and compressing the thickness of the double layer [66]. The valence, hydration state, and chemical structure of ions were all thought to be relevant to the competitive uptaking between acid anions and chromium anions [67]. Though since Cl^−^ was recognized to have a minor affinity through outer-sphere complexes with binding surfaces, its competitive influence on the absorption of Cr(VI) was negligible. The co-existence of SO_4_^2−^ in the solution was seen to have a more important impact on the Cr-uptake process (VI). Monovalent anions Cl^−^ had lower ion energy and electrostatic adsorption than dianions and trivalent anions. Because SO_4_^2−^ had a greater ability to compete for the binding of positively charged surfaces, the adsorption of HCrO_4_^−^ was diminished. SO_4_^2−^ may also respond with positively charged interfering cations upon the TIHP surface, limiting the number of active sites of adsorption that are left as shown in Figure 14.

To evaluate an adsorbent, it is important to consider its adsorption capacity and its desorption properties. Having greater desorption properties in addition to larger absorption capacity is critical for an optimal adsorbent since this lowers both its overall price and its environmental impact. Because of this, some potential reagents were selected for Cr(VI) desorption. A cost-effective and non-damaging reagent is ideal, and some of these substances have proven their value in previous adsorption investigations employing Cr(VI) [68].

### 3.3. Regeneration and Desorption Studies

As part of the regeneration studies, Erlenmeyer flasks filled with 100 mL of 250 mg/L Cr(VI) solution also contained 0.08 g/L of TIHP. The adsorbent was collected together and the adsorption capacity was evaluated after 45 min of equilibration at room temperature. Adsorbent surfaces were rinsed three times and followed by drying in an oven at 70 °C to remove any remaining metal ions from the surface.

Metal ions were removed from the adsorbent using four different agents: de-ionized water, 0.5 M NaOH, 0.5 M HCl and 0.5 M H_2_SO_4_, and 0.5M EDTA. Only 20 mL of the desorption agents Erlenmeyer flannels were used in this experiment, which was conducted for 30 min at 120 rpm and 27 ±  0.5 °C. After equilibration, Whatman type 42 filter paper was applied to filter the adsorbate-adsorbent mixtures. Using a UV-VIS spectrophotometer, diphenyl-carbazide was used to measure the concentration of Cr(VI) ions in the filtrate [69].

**Figure 15:**
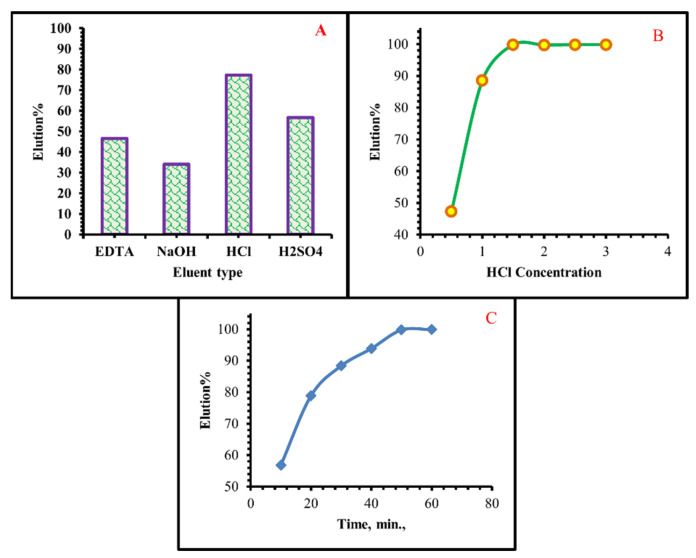
(**A**) Elution results of Cr(VI) from TIHP Surface at various eluting agents (**B**) At different HCl Concentrations (**C**) At the different times.

According to Figure 15, 77.3 percent of the Cr(VI) adsorbed on the adsorbent surface was recovered using HCl. For Cr(VI) ion, the desorption percentages of 0.5 M NaOH, 0.5 M H_2_SO_4_, and 0.5 M EDTA were 34.1%, 56.7%, and 46.5%, respectively. To Cr(VI) ions removed from the TIHP surface, a 0.5 M solution of HCl was chosen as a desorption reagent.

The desorption of Cr(VI) ions employing HCl as a desorption agent is shown in Figure 15, instead of chemical sorption, this effect could be explained by an ion-exchange type interaction [70]. To recover metals and regenerate the exhausted sorbent [71], desorption is an important technique.

To determine the ideal HCl concentration for recovering Cr(VI) from loaded TIHP, several acid concentrations were tried on a sample of 0.08 g TIHP for 30 min at room temperature in 20 mL of various HCl concentrations. For the aim of determining the optimal elution time for maximum elution efficiency, the obtained data in Figure 15 reveal that 1 M HCl is optimal with 50 min elution duration.

### 3.4. Suggested Mechanism of Cr(VI) Ions Adsorption

**Scheme 2:**
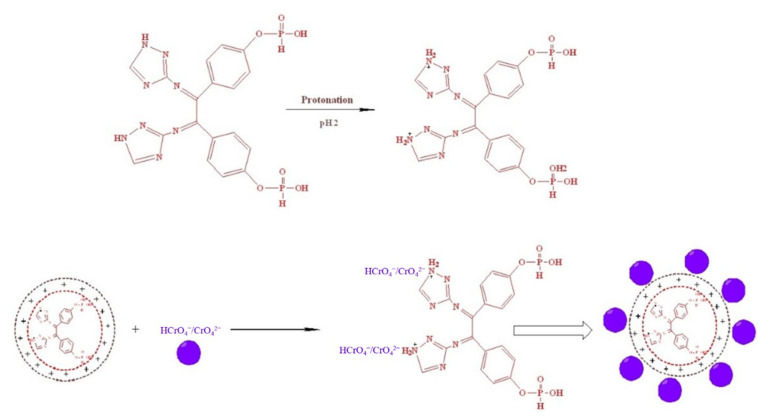
Mechanism of Cr(II) ions removal by TIHP.

The behavior of Cr(VI) adsorption by TIHP at low pH may be related to the presence of a large quantity of H^+^ ions at low pH, which in turn neutralize the adsorbent, which has a negatively charged surface, reducing hindrance to chromate ion diffusion. Additional positively charged adsorbent surface TIHP, thus resulting in an improved binding efficiency between the TIHP and the chromate ions, is seen in Scheme 2 [72].

The rise in pH leads to the decrease in the concentration of H^+^ ions and a gradual increase in the OH^−^ which starts to compete with Cr(VI) species (CrO_4_^2−^) for the presented adsorption sites and so the adsorption of Cr(VI) reductions. For pH > pH_pzc_, a rise in electrostatic repulsion releases the adsorbed HCrO_4_^−^ and CrO_4_^2−^ into the solution [73].

### 3.5. Comparison Study of the Cr(VI) Adsorption Abilities by TIHP with the Other Different Adsorbents

There are clear advantages to using TIHP for adsorption over other adsorbents, as demonstrated in the experiment with Cr (VI) ions (Table 6), which compared the adsorption efficiency of various materials. The adsorption capability of TIHP for Cr(VI) was found to be 307.07 mg∙g^−1^, which is far higher than the capacity of the majority of them. Additionally, the maximum active adsorbent for the adsorption of chromium ions from aqueous solution TIHP conducted a comparison study of the calculated capacities of Cr(VI) with various other adsorbents.

### 3.6. Application on Real Samples

Chromium salts (Chromium sulphate salt (Cr_2_(SO_4_)_3_) utilized during the traditional tanning process yield two types of chrome, namely; hexavalent chromium and trivalent chromium. In Egypt, tannery effluent is released straight into the main domestic sewage pipeline without being properly treated, which causes issues for the sewer system and wastewater treatment plants [88]. In tannery effluent, large levels of contaminants with low biodegradability provide a significant technological and environmental issue. Two samples were collected and were subjected to analysis (Table 7). To assess chromium adsorption and recovery, first of all, Cr(III) was subjected to an oxidized process of Cr(VI) with hydrogen peroxide (30%), according to

$$2{\mathrm{CrO}}_{2}^{-}+3H_{2}O_{2}\to 2{\mathrm{CrO}}_{4}^{2-}+{\mathrm{OH}}^{-}+H_{2}O$$

The optimum conditions for adsorption and elution of Cr(VI) from the working tannery solution were applied using 500 mL of the sample, 2 g of TIHP, pH 2, and 40 min at ambient temperature. Following the completion of the uptake process, TIHP was separated and the Cr(VI) amount was recovered using 1 M HCl for 50 min. The Cr(VI) solution was then subjected to precipitation via the reduction method, which involves converting Cr(VI) to Cr(III) using Na_2_SO_3_ at pH 2 as the reducing agent, then adding ammonia solution drop by drop, which reacts with Cr(III) ion and precipitates as grey-green Cr(III) hydroxide

$${\mathrm{Cr}}^{3+}\left(\mathrm{aq}\right)+3{\mathrm{NH}}_{3}\left(\mathrm{aq}\right)+3H_{2}O\to \mathrm{Cr}{\left(\mathrm{OH}\right)}_{3}\left(S\right)$$

**Figure 16:**
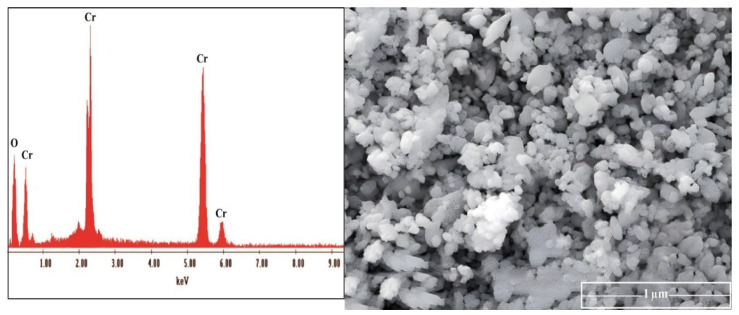
EDAX and SEM of the prepared chromium oxide from tannery wastewater.

Finally, the precipitated chromium obtained product was characterized using EDX, and SEM Figure 16.

## 4. Conclusions

A novel synthetic of phosphorylated Schiff base, 4-((1*E*)-1,2-bis ((1*H*-1,2,4-triazol-3-yl)imino)-2-(4-((hydroxyhydrophosphoryl)oxy)phenyl)ethyl)phenyl hydrogen phosphonate TIHP, was synthesized using an effective substitutional technique compared to the conventional methods and was utilized to uptake Cr(VI) ions. Results show that TIHP has an excellent Cr(VI) adsorption presentation and that the optimum Cr(VI) adsorption efficiency of TIHP is 307.07 mg∙g^−1^. To achieve the batch technique’s optimal settings, we used a 20 mL solution volume containing 1250 mg/L Cr(VI) stirred with 0.08 g of TIHP dosage for 45 min at room temperature with a pH 2 solution. As an alternative, the observed kinetic data agree well with the top model for consideration and suggests the adsorption mechanism of the pseudo-second-order kinetic model. The Langmuir and Freundlich isotherm models were also used to evaluate the equilibrium data. Based on the data, the Freundlich model was found to be the best way to explain how adsorption works. Because of the negative values of both ΔG and ΔH, exothermic and spontaneous Cr(VI) adsorption was possible. It was found that the Cr(VI) ion adsorption on TIHP is very efficient, with a high uptake efficiency from samples of tannery wastewater. EDAX and SEM were utilised to characterize and confirm the high purity of the Cr(VI) product that was isolated from the tannery wastewater.

## Figures and Tables

**Table 1:** THI and THIP characteristics; surface area and pore size.

Adsorbant	BET Surface Area (m^2^/g)	Total Pore Volume (cm^3^/g)	Average Pore Diameter (nm)
THI	37.2	0.189	2.3
THIP	68.4	0.143	1.28

**Table 2:** Kinetic factors controlling Cr(VI) adsorption upon TIHP.

1st Ordered Kinetic				2nd Ordered Kinetic		
**K_1_**	**q_(max)cal_**	**R^2^**	**q_max(exp)_**	**K_2_**	**q_(max)cal_**	**R^2^**
0.11469	143.549	0.9639	62.44	0.001533	70.922	0.994

**Table 3:** Parameters of Langmuir and Freundlich models of Cr(VI) adsorption upon TIHP.

Langmuir					Freundlich			
**K_l_**	**q_max(cal)_**	**χ^2^**	**R^2^**	**q_max(exp)_**	**K_f_**	**1/n**	**χ^2^**	**R^2^**
0.01678	320.226	57.283	0.895	307.07	33.51	2.6647	6.2197	0.969

**Table 4:** Thermodynamic factors for Cr(VI) ions adsorption by TIHP.

ΔH^°^ (kJ∙mol^−1^)	ΔS^°^ (J mol^−1^∙K^−1^)	ΔG^°^ (kJ∙mol^−1^)				
−10.883	9.811	298 K	308 K	318 K	328 K	343 K
		−2.935	−3.033	−3.131	−3.229	−3.376

**Table 5:** Comparison of the physico-chemical properties of the cations.

Ions	Pb^2+^	Fe^3+^	Al^3+^	Cd^2+^	Cu^2+^	Zn^2+^
Ionic radius	119	64.5	53.5	95	73	74

**Table 6:** Comparison study of the Cr(VI) adsorption capabilities by TIHP with the other different adsorbents.

Adsorbent	Capacity of Adsorption, mg/g	References
Apricot stone	262	[74]
Rubber wood sawdust	65	[75]
Almond shell	167.5	[76]
Sterculia guttat shell	45.5	[77]
Chitosan beads	76.92	[78]
Schiff base-chitosan grafted multiwalled carbon nanotubes	95.0	[79]
ethylenediamine-modified cross-linked magnetic chitosan resin	51.0	[80]
Chitosan flakes	34.40	[81]
Zr(IV)-impregnated collagen fiber	27.56	[82]
Trimethoxysilyl group terminated poly(1-vinylimidazole)- modified-chitosan composite	151.5	[83]
Green rust functionalized geopolymer of composite cementitious materials	55.01	[84]
Fe3O4 loaded on activated carbon	15.24	[85]
Natural zeolite coated with magnetic nanoparticles	43:933	[86]
Date palm empty fruit bunch wastes	70.49	[87]
THIP	307.07	This study

**Table 7:** Physicochemical analysis for real tannery wastewater samples.

Parameters	Sample 1	Sample 2
pH	8.4	8.9
Chemical oxygen demand (COD) (mg/L)	7560	7140
Biological oxygen demand (BOD) (mg/L)	5950	6050
Chromium (mg/L)	817	1170
SO_4_^2−^ (mg/L)	1826	2425
Cl^−^ (mg/L)	3014	4768
Ammonia nitrogen (mg/L)	50	48
Phosphorous (mg/L)	61	58
Total suspended solids (TSS) (mg/L)	2930	3650

## Data Availability

The data presented in this study are available upon request from the corresponding author.

## Supplementary Materials

The following supporting information can be downloaded at: https://www.mdpi.com/article/10.3390/molecules27165087/s1, Figure S1: BET surface area of (A) TIH and its Pore volume, B) THIP and its Pore volume; Figure S2: (A1&A2) ^13^C-NMR analysis of TIHP and (B) ^31^P-NMR analysis of TIHP.; Figure S3: The intraparticle diffusion kinetic model of the adsorption process of Cr(VI) by TIHP.

## Sample Availability

The data presented in this study are available on request from the corresponding authors.
