# Supplementary material for: A Novel Triazole Schiff Base Derivatives for Remediation of Chromium Contamination from Tannery Waste Water
Source: Molecules. 2022 Aug 10;27(16):5087. doi: 10.3390/molecules27165087 (PMC9415994; doi:10.3390/molecules27165087)
Supplement: Supplementary file 1 [file molecules-27-05087-s001.zip › molecules-1839046-supplementary.pdf]

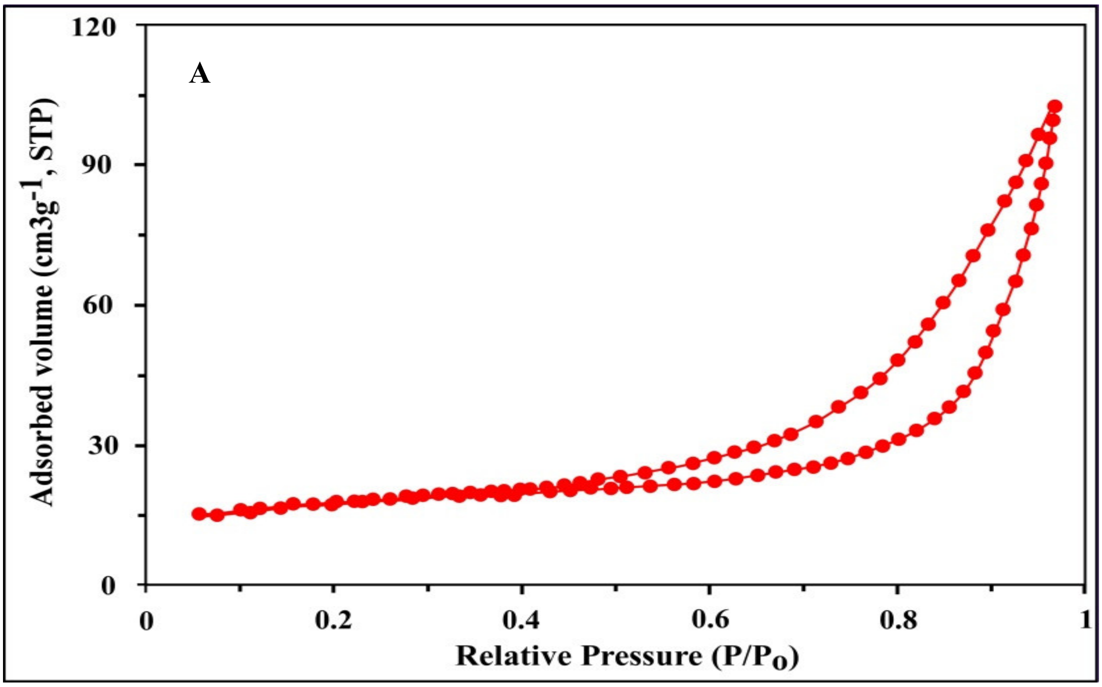
A
Adsorbed volume (cm3g-1, STP)
Relative Pressure (P/Po)
120
90
60
30
0
0
0.2
0.4
0.6
0.8
1

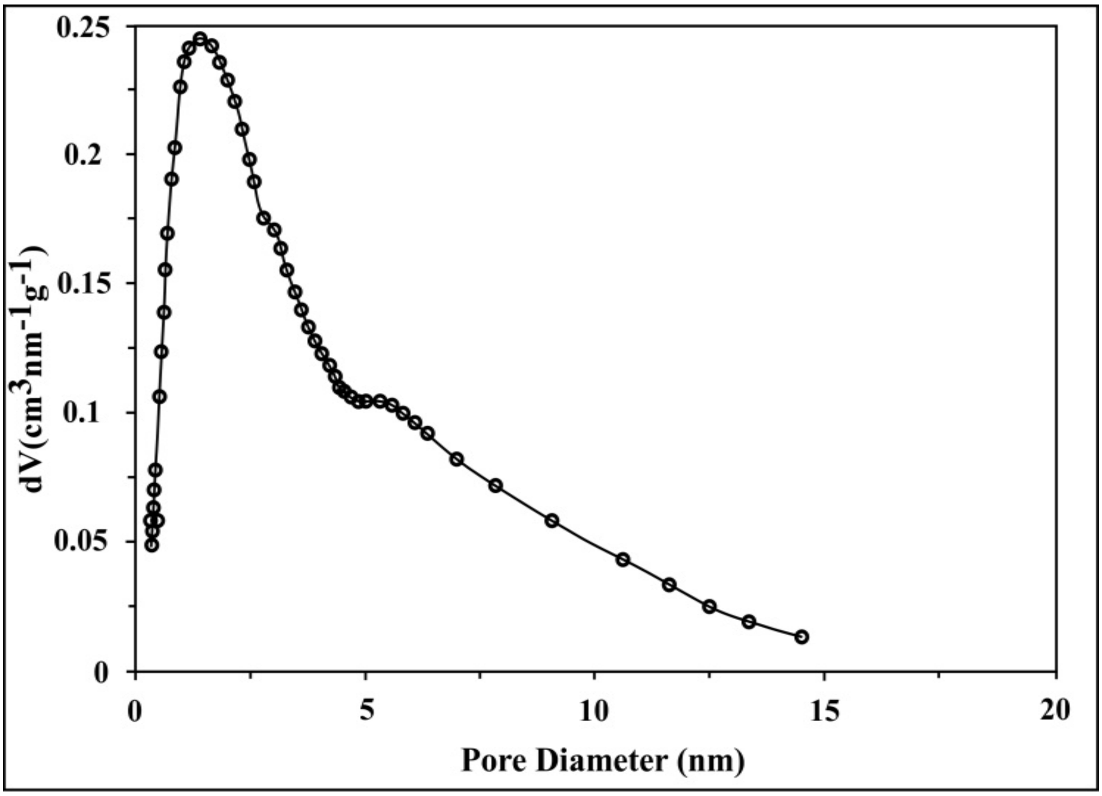
dV(cm3nm-1g-1)
Pore Diameter (nm)
0.25
0.2
0.15
0.1
0.05
0
0
5
10
15
20

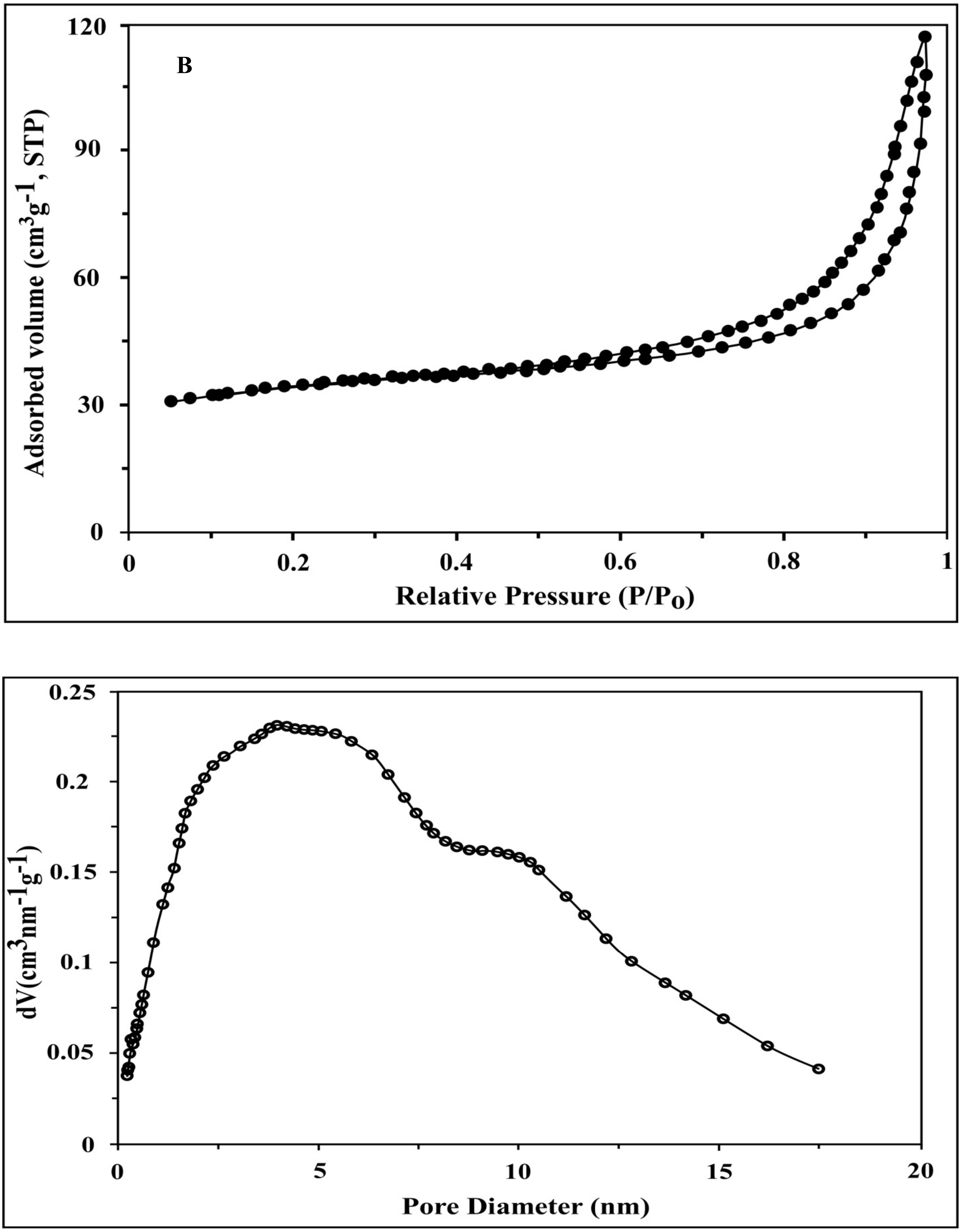

Figure S1. BET surface area of (**A**) TIH and its Pore volume, (**B**) THIP and its Pore volume.

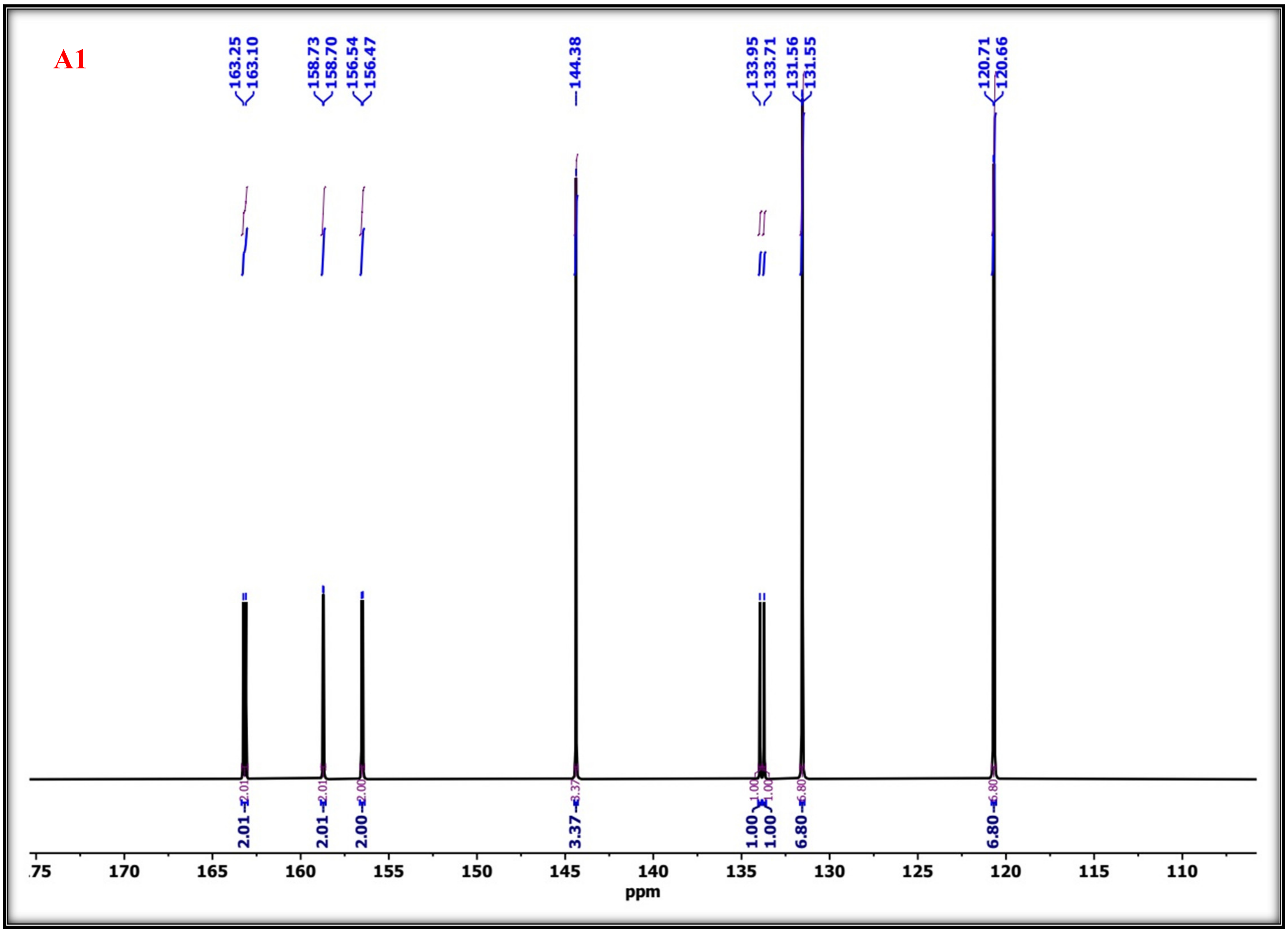
A1
163.25
163.10
158.73
158.70
156.54
156.47
144.38
133.95
133.71
131.56
131.55
120.71
120.66
2.01
2.01
2.00
3.37
1.00
1.00
6.80
6.80
175
170
165
160
155
150
145
140
135
130
125
120
115
110
ppm

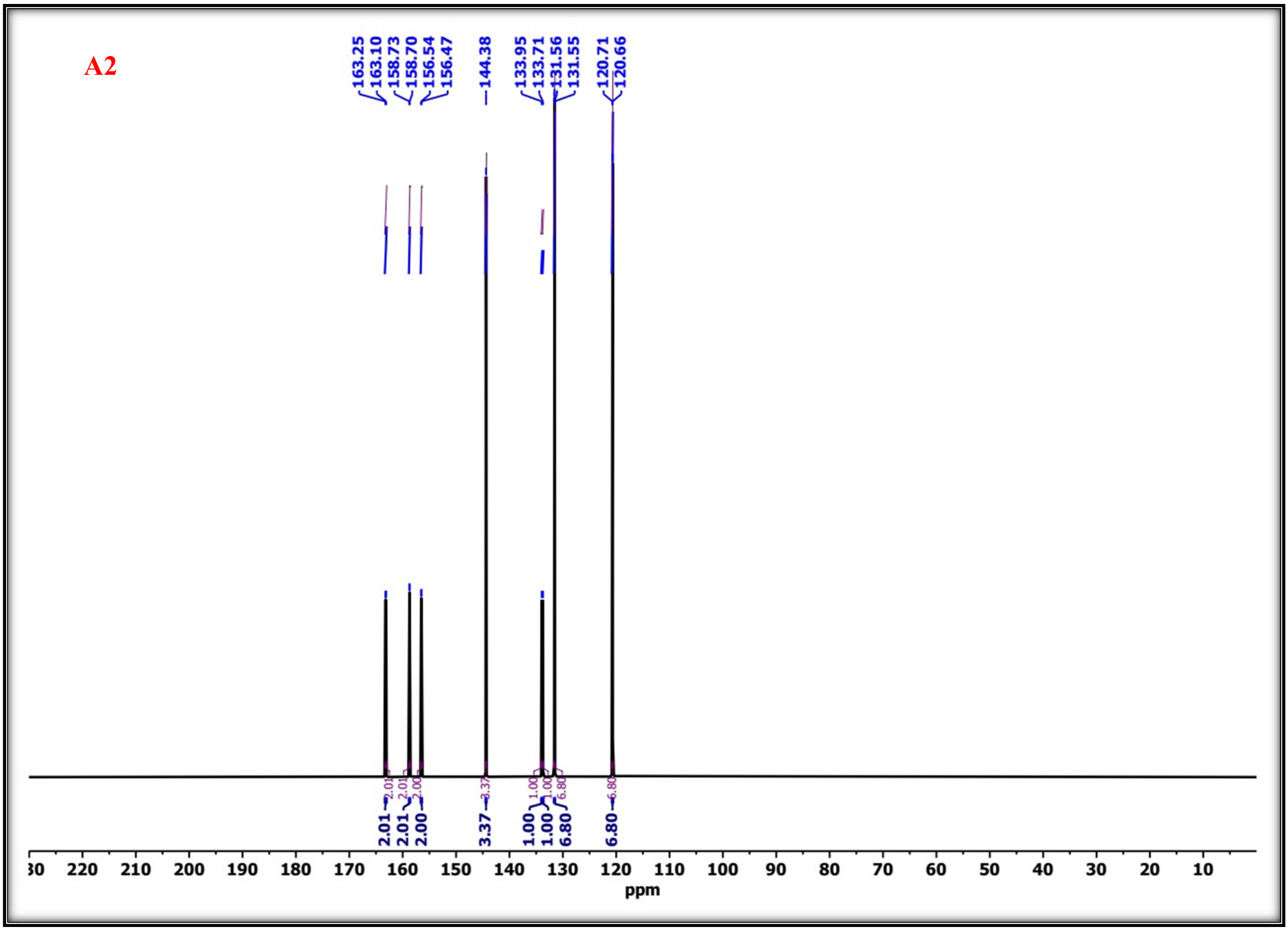

A2
163.25
163.10
158.73
158.70
156.54
156.47
144.38
133.95
133.71
131.56
131.55
120.71
120.66
2.01
2.01
2.00
3.37
1.00
1.00
6.80
6.80
220 210 200 190 180 170 160 150 140 130 120 110 100 90 80 70 60 50 40 30 20 10
ppm

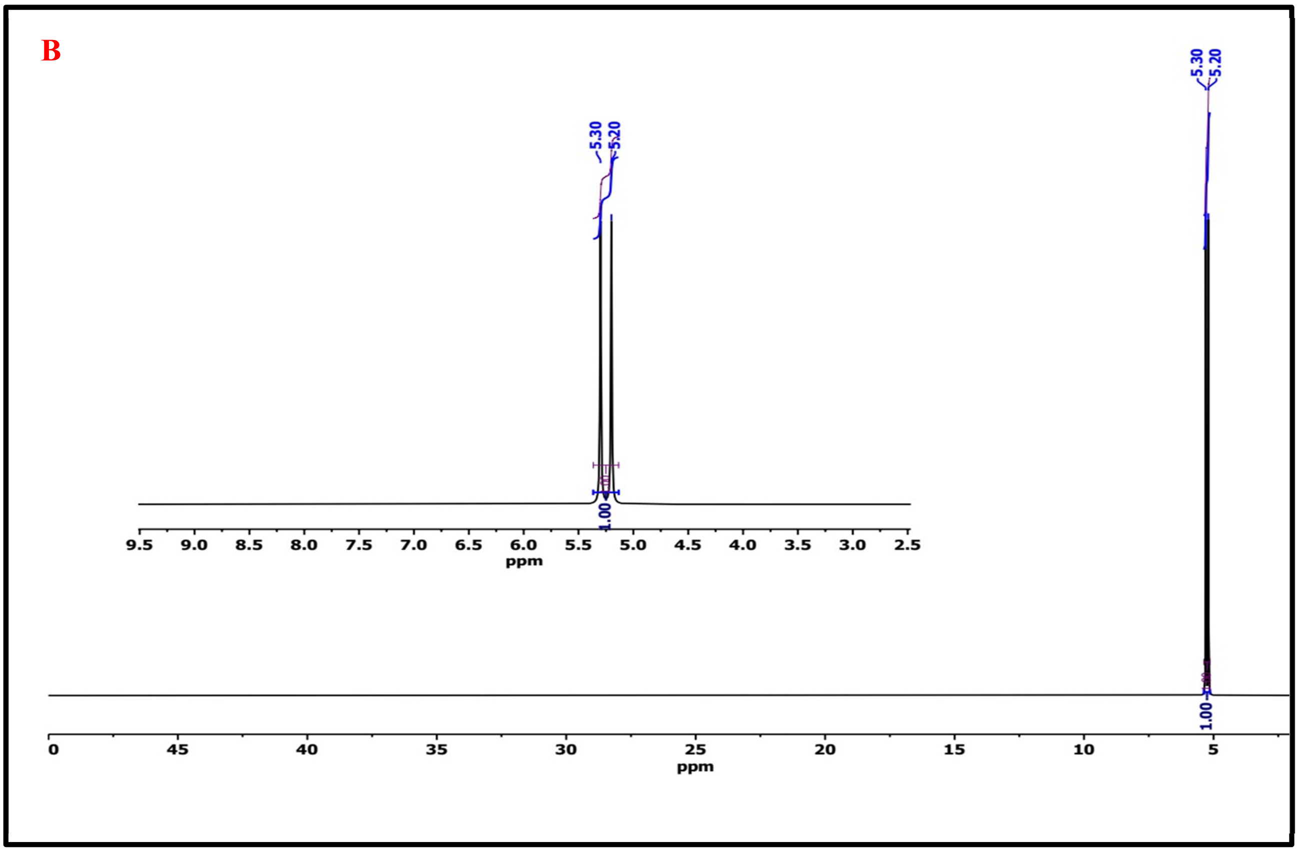

Figure S2. (**A1**,**A2**) ^{13}C-NMR analysis of TIHP and (**B**) ^{31}P-NMR analysis of TIHP.

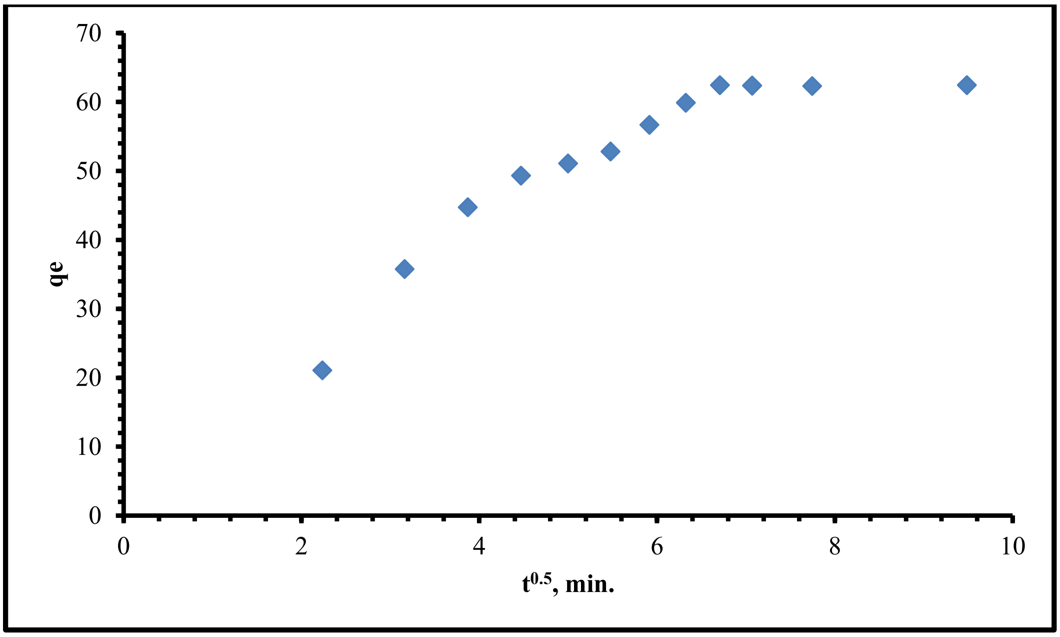

Figure S3. The intraparticle diffusion kinetic model of the adsorption process of Cr(VI) by TIHP.
